# Influence of aging on the behavioral phenotypes of C57BL/6J mice after social defeat

**DOI:** 10.1371/journal.pone.0222076

**Published:** 2019-09-03

**Authors:** Hiroaki Oizumi, Nae Kuriyama, Sachiko Imamura, Masahiro Tabuchi, Yuji Omiya, Kazushige Mizoguchi, Hiroyuki Kobayashi

**Affiliations:** 1 Tsumura Kampo Research Laboratories, Tsumura & Co., Ibaraki, Japan; 2 Center for Advanced Kampo Medicine and Clinical Research, Juntendo Graduate School of Medicine, Tokyo, Japan; Oregon Health and Science University, UNITED STATES

## Abstract

Depression and anxiety are common psychiatric disorders that can occur throughout an individual’s lifetime. Numerous pathways underlying the onset of these diseases have been identified in rodents using a social defeat stress protocol, whereby socially defeated individuals exhibit depression- and/or anxiety-like phenotypes that typically manifest as social avoidance behavior. However, most studies in this field have been conducted using young adult mice; therefore, information about social defeat stress-related behavioral phenotypes in older mice is limited. In this study, we exposed groups of young adult (8–16 weeks old) and aged (24 months old) C57BL/6J mice to mild social defeat stress by challenging them with aggressive CD1 mice while restricting the intensity of aggression to protect the animals from severe injuries. We then identified stress-induced behavioral changes and compared their expression between the age groups and with a non-defeated (non-stressed) control group. We found that the stressed mice in both age groups exhibited similar reduced social interactions that were indicative of increased social avoidance behavior. Moreover, unlike the young stressed and control groups, only the aged stressed group showed a reduced preference for sucrose, which was correlated with social avoidance behavior. Also, the aged stressed mice exhibited an attenuated defeat-induced increase in water intake. These findings reveal that aging alters behavioral phenotypes after social defeat and that the hedonic behavior of aged mice is more vulnerable to social defeat compared with younger mice.

## Introduction

Depression and anxiety are common psychiatric disorders that have a variety of etiologies, including both environmental and social stressors as well as biological vulnerability in many cases. Depression is characterized by core symptoms, such as depressive mood, anhedonia, and apathy, whereas anxiety disorders are characterized by elevated feelings of fear and worry at a general (generalized anxiety disorder and panic disorder) or specific (social anxiety disorder and phobia) level. Depression and anxiety most commonly occur in young or middle-aged people; however, they are frequently identified throughout the human lifespan, including in elderly individuals [1] who may experience distinct symptoms that require different treatment regimens than younger patients [2, 3]. Also, subthreshold depression is as prevalent as clinically significant depression among elderly people, with 9–10% of the community-dwelling elderly individuals suffering from this condition, indicating a strong need for mental care in the elderly [4]. Consequently, measures should be taken to prevent mental disorders from occurring in elderly people and treatment programs should be established for psychiatric disorders in older people to prepare for the growing aging populations in many parts of the world, particularly in developed countries. Thus, an animal model of psychiatric disorders in the elderly is required to evaluate drug efficacy in aged individuals.

The repeated social defeat stress model in rodents, whereby dominant individuals attack submissive ones as a result of a perceived challenge, is thought to mimic chronic social stress in human society [5]. Social defeat stress has been shown to cause depression- and anxiety-like behaviors in subordinate mice [6, 7]. Notably, a subpopulation of the defeated mice displays social avoidance behavior when presented with an unfamiliar conspecific, whereas the rest of the defeated individuals do not display this behavior [7–9]. Interestingly, this social avoidance behavior is well-associated with other behavioral changes, such as anhedonia and changes in appetite, indicating that it can serve as a barometer of behavioral abnormalities in socially defeated mice [7]. Furthermore, it has been shown that the social avoidance behavior can be reversed by the chronic, but not acute, administration of antidepressants, similar to the therapeutic delay in treating human depression with these drugs [10]. Consequently, the repeated social defeat model is considered to have constitutive, face, and predictive validities as a model of major depression in humans, resulting in it being a common animal model for affective disorders. However, little is known about the repeated social defeat model when used with aged animals.

Several reports have indicated that aging is related to changes in the behavioral and molecular responses to social defeat. For example, in an open field test, Kinsey et al. [11] demonstrated that after social defeat, middle-aged subordinate mice (14 months old) showed increased anxiety-like behavior and enhanced inflammatory responses including increased interleukin 6 (IL-6) and tumor necrosis factor alpha (TNF-α) levels compared with young subordinates (2 months old), suggesting that aging exacerbates vulnerability to social stress in mice [11]. However, these researchers precluded the use of aged mice out of concern that they might be severely hurt by the aggressors, and did not confirm whether the subordinate mice exhibited social avoidance behavior [11] that is correlated with other psychiatric abnormalities and a susceptibility to social stress in rodents [7, 11].

Therefore, in the present study, we investigated the effects of social defeat on the behavioral phenotypes of aged mice by exposing young adult (8–16 weeks old) and aged (24 months old) mice to mild social defeat stress (MSDS) and comparing the behavioral phenotypes of the groups with respect to social interaction (SI) and sucrose preference. Also, we monitored the physiological responses of the mice in each group by measuring their body weights and liquid intakes during the stress period. We restricted the stress to a mild level compared with typical social defeat stress to prevent the aged subordinate mice from suffering severe wounds.

## Materials and methods

### Animals

Two groups of male C57BL/6J mice were used during the social defeat period: 8–16-week-old (young) mice and 24-month-old (aged) mice. The mice were group-housed in standard cages (3–5 mice per cage) and acclimatized to a temperature- and humidity-controlled room for at least 7 days prior to being moved to a new cage and presented with an unknown CD1 mouse to induce social defeat (see below). The mice were maintained under a 12-h light/dark cycle (lights on at 07:00 AM) with *ad libitum* access to food and water. The aged group consisted of C57BL/6J mice weighing 28–40 g without any apparent injuries or loss of voluntary movements. Both young and aged C57BL/6J mice were randomly divided into the following 4 groups on the day of social defeat (Day 1) as follows: young non-defeated (young control, n = 22); young defeated (young stressed, n = 22); aged control (n = 22); and aged stressed (n = 29). Any mice that exhibited an apparent reduction in locomotion during the experiments were humanely euthanized and removed from the study. Euthanasia was performed by trained staff using cervical dislocation.

Retired male CD1 breeder mice aged 6–12 months were used as aggressors and housed individually until screening. Screening for aggressive CD1 mice was performed according to previous reports [12]. Briefly, a screening session was carried out once per day on 3 consecutive days and CD1 mice that initiated an attack bite on unfamiliar C57BL/6J screeners within 60 s in at least 2 consecutive sessions were selected as aggressors.

All mice used in this study were purchased from Charles River Co. Ltd. (Tokyo, Japan). This study was conducted in accordance with the guidelines for the care and use of experimental animals of Tsumura & Co. (Ibaraki, Japan) and the protocol was approved by the experimental animal ethics committees of Tsumura & Co. (approval no: 17–026).

### Social defeat

The social defeat stress protocol was carried out using the previously reported resident/intruder paradigm [7, 10, 12] with some modifications. Briefly, each experimental mouse was placed in a home cage housing a CD1 (aggressor) mouse and experienced attack bites (a defeat episode). After the defeat episode, both mice were separated by a perforated divider and housed in the same cage for 24 h to expose the subordinate mouse to sensory stress without physical contact. The 24-h defeat cycle was repeated for 7 consecutive days, changing the resident-intruder pair each day to prevent habituation. To limit stress and physical injury to the subordinate mice, we modified the previously reported protocol by limiting the number of defeat cycles to 7 (versus 10) and limiting the duration of the defeat episodes to 5 min on Day 1 and 3 min on Days 2–7 (versus 10 min/day) [7, 10, 12]. We set a longer duration for defeat for Day 1 because our preliminary experiments showed that the aggressors took longer to start an attack on Day 1 than on subsequent days. Furthermore, the defeat episode was stopped regardless of its duration when the experimental mouse had been bitten 3 times. As a control, non-defeated (non-stressed) mice were pair-housed with other C57BL/6J control mice in divided cages with no physical contact between the mice and the pairings were changed daily. The latency, number, and duration of attacks were recorded for every defeat episode. In addition, because repeated social defeat stress often causes changes in body weight and liquid intake, both the water containers and mice were weighed each day before the defeat episode. After the last defeat episode, all experimental and control mice were housed individually.

### Social interaction test

SI testing was performed on the day after the last defeat episode (i.e., Day 8) following previously reported methods [7, 10, 12] with slight modifications. Each mouse was placed in an open field (50 cm wide × 50 cm deep × 25 cm high), that had a wire-mesh enclosure (10 cm wide × 10 cm deep × 10 cm high) on 1 end. Dim red lighting (30 lux) was used and the mouse was allowed to move freely in the field. Each test consisted of 2 sessions lasting 150 s: a target-absent session, in which the wire-mesh enclosure was empty, and a target-present session, in which a social target mouse was enclosed within the wire-mesh enclosure. Aggressive CD1 mice that were novel to the C57BL/6J mice were used as social targets.

The exploratory behaviors of the mice were recorded with a charge-coupled device (CCD) camera (Sony, Tokyo, Japan) and analyzed with video tracking software (LimeLight 2; Actimetrics, Illinois, USA). The amount of time each mouse spent in a rectangular area (26 cm wide × 18 cm deep) surrounding the enclosure, defined as the interaction zone (IZ), was determined and the SI ratio was calculated as the ratio of the time a mouse spent in the IZ when the target was present to that when the target was absent; these were considered to be indicators of social avoidance behavior. Additionally, the distance each mouse traveled in the absence of the target was measured to calculate locomotor activity. After the target-present session, the field was cleaned with wet paper towels and the test session was repeated with a different mouse.

### Sucrose preference test

Stressed mice exhibit a lower preference for sucrose solution over tap water than non-stressed control mice [7, 13]. Therefore, the sucrose preference test was conducted following previously published methods [7]. Briefly, the mice were allowed to freely choose to drink from 2 water bottles containing either tap water or a 1% sucrose solution. These bottles were placed on each mouse cage after the SI test (i.e., on Day 8) and the mice were allowed to acclimatize to them for 24 h. After the acclimatization period, the weight of each bottle was measured each day for 4 consecutive days (i.e., Days 9–12), switching the position of each bottle (left vs. right) every day to avoid position bias. The daily sucrose preference was then calculated as follows: (weight of sucrose solution consumed) / (total weight of liquid consumed) × 100. The daily sucrose preference and total weight of liquid consumed per body weight over the 3 days was averaged for each animal.

### Statistical analyses

All 2-group comparisons were made using Student’s or Aspin–Welch’s *t*-tests. Data from the SI and sucrose preference tests were analyzed using 2-way analysis of variance (ANOVA) followed by a Bonferroni test. Survival curves of the aged groups were analyzed with a log rank test. Stressed mice in the young and aged groups were split into 2 subpopulations based on the results of the SI test (stress resilient [SI > 1.0] and stress susceptible [SI < 1.0]) as described previously [7, 12] and the frequency distribution of these subpopulations was analyzed using a *chi*^2^ test. Chronological changes in body weight and water intake were analyzed with 2-way repeated measures ANOVA followed by a Bonferroni test. Statistical significance was defined as *p* < 0.05.

## Results

### Effect of the mild social defeat stress protocol on young and aged mice

**Fig 1:**
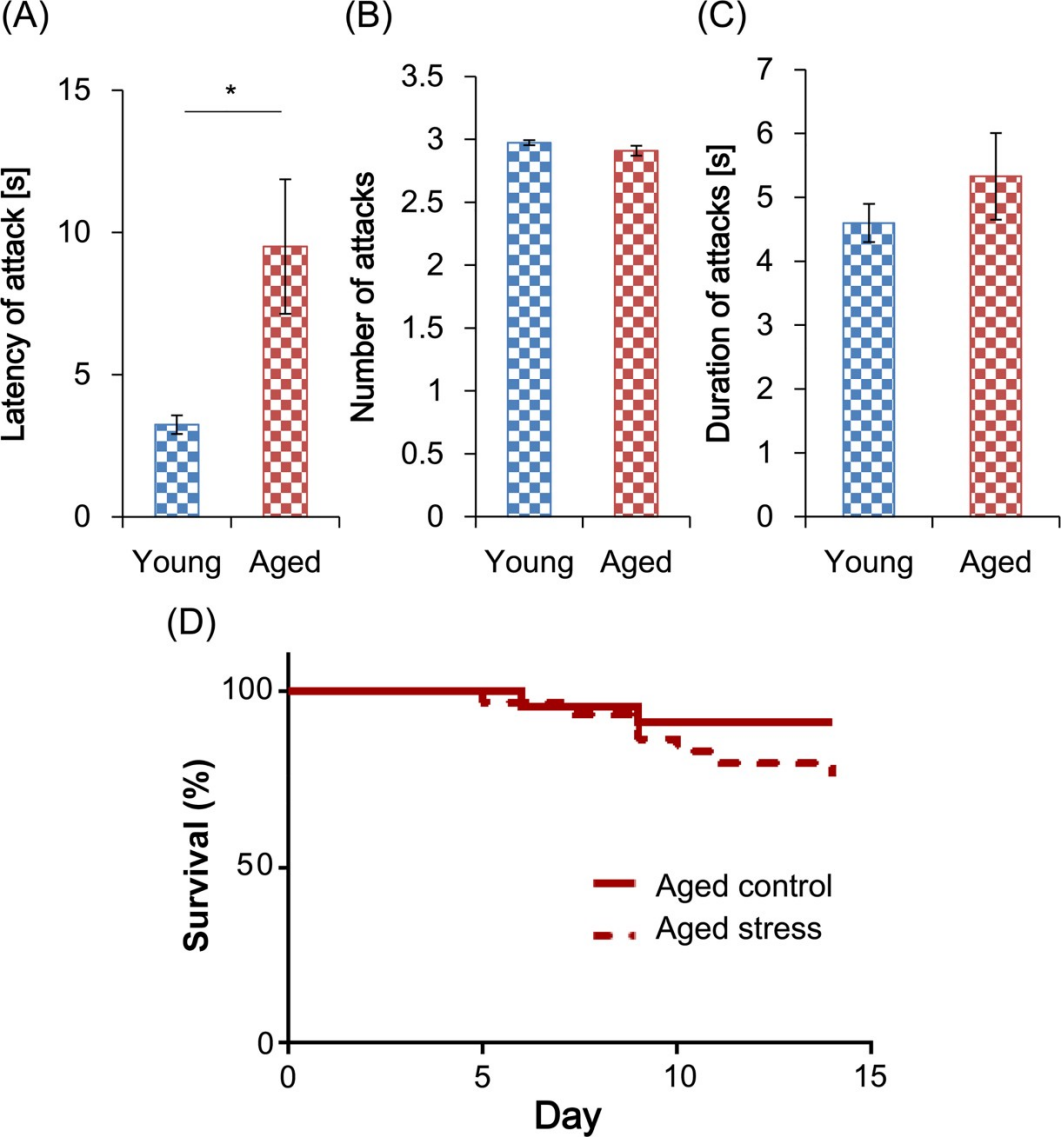
Latency and number of attacks by CD1 mice on experimental mice. (A) The latency to the first attack, (B) number of attacks, and (C) duration from the first to the third attack are shown. Values during each defeat session were averaged for each subordinate mouse and compared between age groups. Data are presented as means ± standard error of the mean (SEM) (young, *n* = 22; aged, *n* = 22). *: *p* < 0.05 (Aspin–Welch’s *t*-test). (D) Kaplan–Meier survival curves from the aged groups during Days 1–14 are shown.

None of the experimental mice experienced severe surface wounds during the repeated social defeat period, although some individuals occasionally received minor bite wounds on their tails. Attack latency by the CD1 mice was significantly longer in the aged group than in the young (*t*_42_ = 2.624, *p* < 0.05; Fig 1A) but both stressed groups experienced a similar number of attacks (*t*_42_ = 1.438, *p* = 0.1604) and comparable attack durations (*t*_42_ = 1.022, *p* = 0.3155) (Fig 1B and 1C). Bite locations were also similar between aged and young mice, with both subordinate groups being bitten on the dorsal surface of the body from the back to the tail (data not shown). The survival curves of the aged groups during Days 1–14 were not significantly altered by MSDS exposure (*chi*^2^_1_ = 1.786, *p* = 0.1814; Fig 1D), with approximately 10% and 25% of control and stressed mice dying by Day 14, respectively. To minimize the effect on the behavioral tests of the systemic poor health that some aged mice in both groups exhibited as a consequence of aging, we limited analyses to only the individuals that survived for 7 days after MSDS exposure and excluded all data from the dead mice from the study.

### Social avoidance in young and aged stressed mice

**Fig 2:**
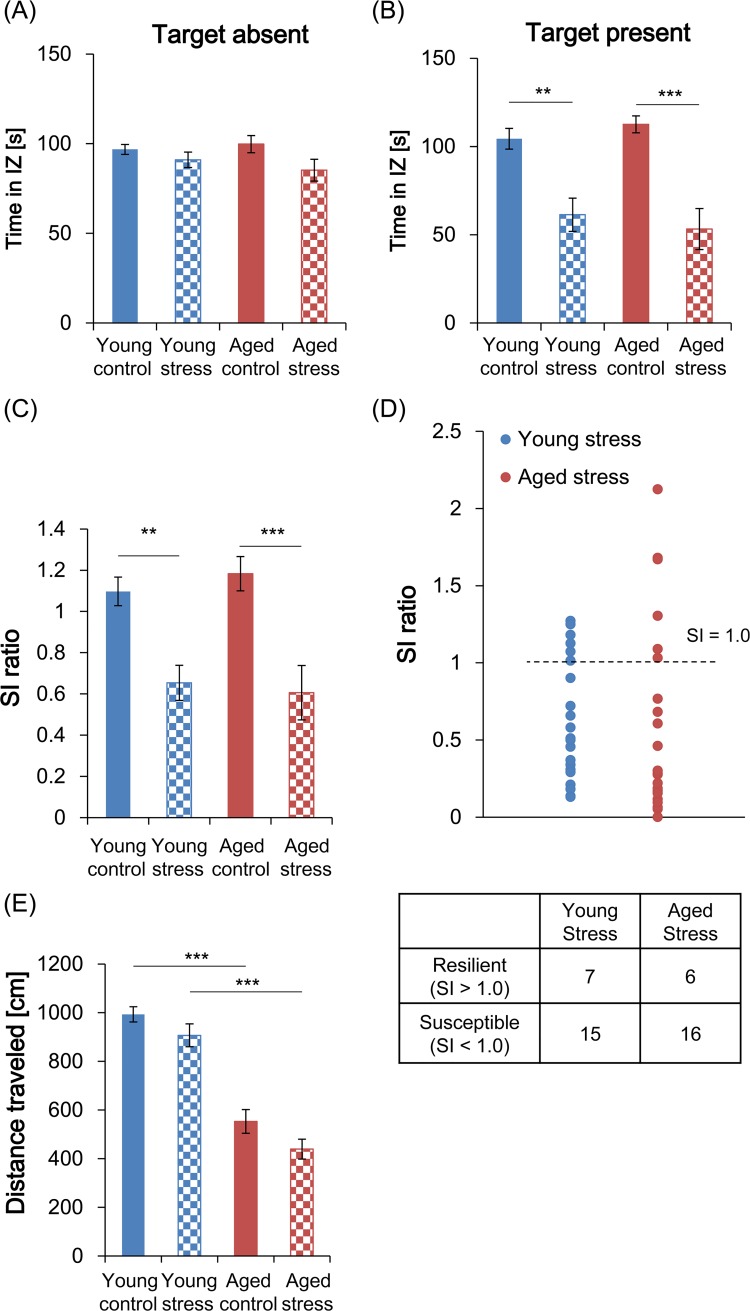
Social avoidance behavior in the social interaction (SI) test. (A–C) SI was assessed by recording the time spent in the interaction zone (IZ) in the absence (A) or presence (B) of the social target and by calculating the SI ratio (C). (D) The subpopulation of defeated (stressed) mice was identified by the SI ratio and is shown in the contingency table. (E) Locomotor activity was evaluated by measuring the distance traveled during the target-absent test sessions. Data in A–C and E are presented as group means ± standard error of the mean (SEM) (young control, *n* = 22; young stressed, *n* = 22; aged control, *n* = 20; aged stressed, *n* = 22). **: *p* < 0.01; ***: *p* < 0.001 (Bonferroni test).

The time spent in the IZ by each group in the target-absent session was significantly affected by stress (2-way *F*_1,82_ = 4.810, *p* < 0.05), but not by age (*F*_1,82_ = 0.09077, *p* = 0.7640) and the stress × age interaction was not significant (*F*_1,82_ = 0.8817, *p* = 0.3505) (Fig 2A). However, post-hoc multiple comparisons indicated that there were no significant differences between the control and stressed groups in the age-matched comparisons (young: *t*_82_ = 0.8978, *p* = 0.7439; aged: *t*_82_ = 2.188, *p* = 0.0630), or between the young and aged groups in the treatment-matched comparisons (control: *t*_82_ = 0.4455, *p* > 0.9999; stressed: *t*_82_ = 0.8879, *p* = 0.7544) (Fig 2A).

In the target-present session, the time spent in the IZ was significantly affected by stress (2-way ANOVA: *F*_1,82_ = 36.22, *p* < 0.001), but not age (*F*_1,82_ = 1.965e-5, *p* = 0.9965), and the stress × age interaction was not significant (*F*_1,82_ = 0.9178, *p* = 0.3409) (Fig 2B). Post-hoc comparisons indicated that the stressed mice spent significantly less time in the IZ than the control mice in both the young (*t*_82_ = 3.623, *p* < 0.01) and aged (*t*_82_ = 4.874, *p* < 0.001) groups and that there was no significant difference in the time spent in the IZ between stressed mice in the young and aged groups (*t*_82_ = 0.6827, *p* = 0.9935).

Similarly, we found that the SI ratio was significantly affected only by stress (2-way ANOVA: stress: *F*_1,82_ = 28.15, *p* < 0.001; age: *F*_1, 82_ = 0.03928, *p* = 0.8434; stress × age: *F*_1,82_ = 0.4826, *p* = 0.4892), with the stressed mice in each age group exhibiting significantly lower SI ratios than their respective controls (Fig 2C). Post-hoc comparisons indicated that this was the case for both the young (*t*_82_ = 3.301, *p* < 0.01) and aged (*t*_82_ = 4.192, *p* < 0.001) groups, with no significant difference between the stressed groups (*t*_82_ = 0.3554, *p* > 0.9999). A contingency table analysis of the resilient and susceptible subpopulations in the young and aged groups of stressed mice showed that there was no significant difference in the distribution of susceptible mice (*chi*^2^_1_ = 0.1092, *p* = 0.7411).

In the target-absent session, locomotor activity was significantly affected by stress (2-way ANOVA: *F*_1,82_ = 5.582, *p* < 0.05) and age (*F*_1,82_ = 114.9, *p* < 0.001), with no significant stress × age interaction (*F*_1,82_ = 0.1034, *p* = 0.7486) (Fig 2E). Post-hoc comparisons confirmed that the distance traveled was significantly lower in aged mice than in young mice in the treatment-matched comparisons (control: *t*_82_ = 7.263, *p* < 0.001; stressed: *t*_82_ = 7.903, *p* < 0.001) but that there was no significant difference between control and stressed mice in the age-matched comparisons (young: *t*_82_ = 1.461, *p* = 0.2956; aged: *t*_82_ = 1.875, *p* = 0.1286).

### Sucrose preference in young and aged mice

**Fig 3:**
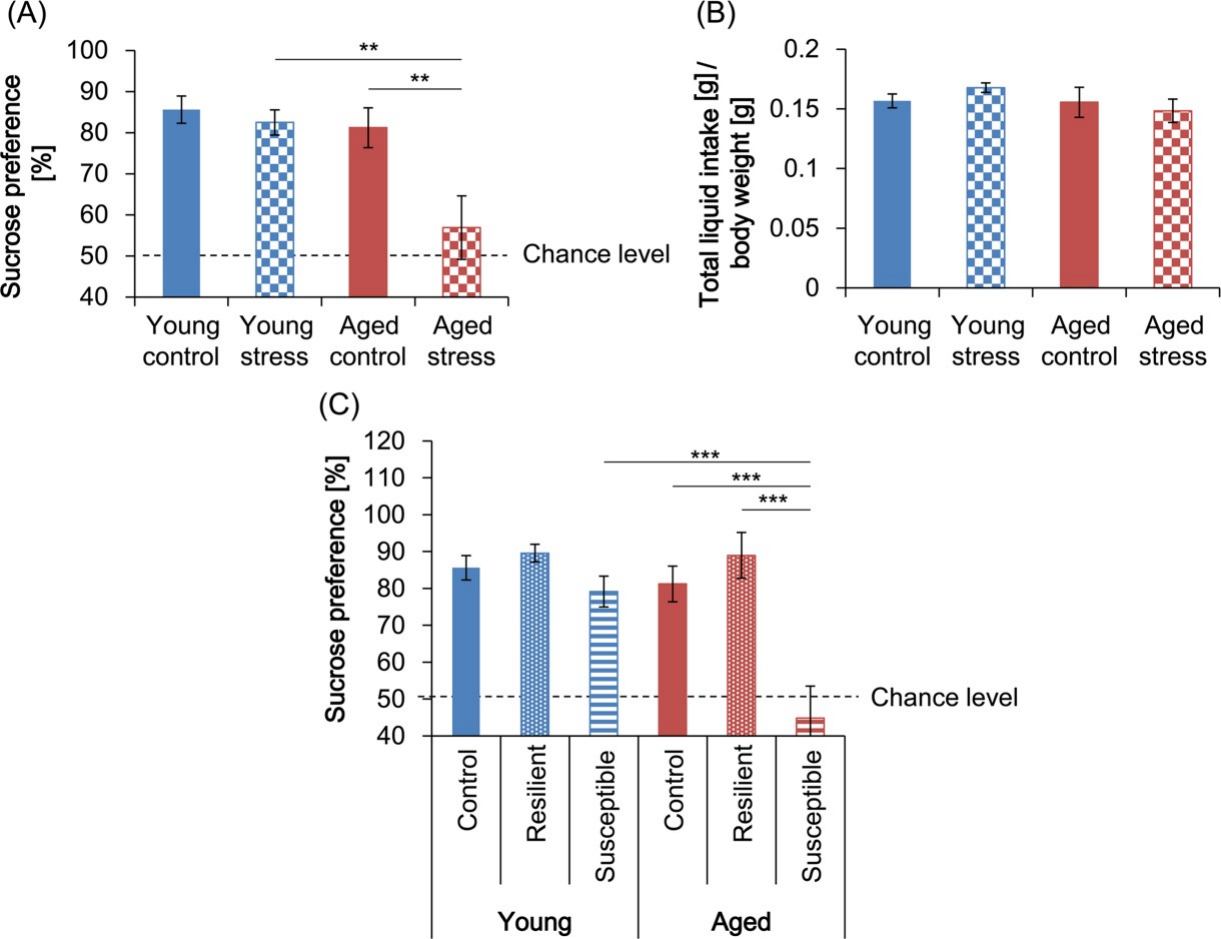
Sucrose preference in young and aged mice after mild social defeat stress (MSDS). (A) The ratio of sucrose intake to overall liquid intake and (B) overall liquid intake per body weight by young and aged mice in the stressed and control groups were measured by placing 2 bottles containing tap water and a 1% sucrose solution, respectively, in each cage and exchanging the bottle positions (left vs right) each day for 3 consecutive days, then averaging the values for each mouse. (C) The sucrose preference in control, resilient, and susceptible subgroups is shown. Data are presented as means ± standard error of the mean (SEM) (young control, *n* = 22; young stressed, *n* = 22; aged control, *n* = 20; aged stressed, *n* = 22; young resilient, *n* = 7; young susceptible, *n* = 15; aged resilient, *n* = 6; aged susceptible, *n* = 16). **: *p* < 0.01; ***: *p* < 0.001 (Bonferroni test).

Sucrose preference was significantly affected by stress (2-way ANOVA: *F*_1,82_ = 7.248, *p* < 0.01) and age (*F*_1,82_ = 8.637, *p* < 0.01); also, there was a significant stress × age interaction (*F*_1,82_ = 4.317, *p* < 0.05) (Fig 3A). Post-hoc comparisons revealed that stressed mice had a significantly lower percentage of sucrose intake than non-stressed controls in the aged group (*t*_82_ = 3.332, *p* < 0.01) but not in the young group (*t*_82_ = 0.4399, *p* > 0.9999). There was also a significant difference in sucrose preference between young and aged stressed mice (*t*_82_ = 3.591, *p* < 0.01), indicating that the susceptibility to social defeat increased with age (Fig 3A).

The total liquid intake (i.e., tap water and sucrose solution) per body weight was not significantly affected by stress (2-way ANOVA: *F*_1,82_ = 0.05502, *p* = 0.8151) or age (*F*_1,82_ = 1.446, *p* = 0.2326) and there was no significant stress × age interaction (*F*_1,82_ = 1.162, *p* = 0.2842; Fig 3B). Bonferroni comparisons indicated that there was no significant difference between the aged and young control mice (*t*_82_ = 0.08707, *p* > 0.9999), between the aged and young stressed mice (*t*_82_ = 1.633, *p* = 0.2128), or between the control and stressed mice in the age-matched comparisons (young: *t*_82_ = 0.9396, *p* = 0.7004; aged: *t*_82_ = 0.5892, *p* > 0.9999).

Since sucrose preference in defeated mice has been shown to be closely associated with the SI phenotype [7], we compared control, resilient, and susceptible subgroups of both ages. Sucrose preference was significantly affected by the SI phenotype (2-way ANOVA: *F*_2,80_ = 10.06, *p* < 0.001) and age (*F*_1,80_ = 6.29, *p* < 0.05) and there was a significant SI × age interaction (*F*_2,80_ = 4.162, *p* < 0.05) (Fig 3C). Post-hoc comparisons indicated that aged susceptible mice had a lower preference for sucrose than aged controls (*t*_80_ = 5.055, *p* < 0.001) and the aged resilient mice (*t*_80_ = 4.296, *p* < 0.001), with no significant difference between the controls and resilient animals (*t*_80_ = 0.7758, *p* > 0.9999). In contrast, this was not the case for the young groups (control vs. resilient: *t*_80_ = 0.4255, *p* > 0.9999; control vs. susceptible: *t*_80_ = 0.8975, *p* > 0.9999; resilient vs. susceptible: *t*_80_ = 1.060, *p* > 0.9999). Moreover, the aged susceptible mice had a significantly lower sucrose preference than their young susceptible counterparts (*t*_80_ = 4.451, *p* < 0.001). These results clearly indicate that the aged mice were more vulnerable to social defeat than the young mice.

### Effect of mild social defeat stress on body weight and water intake in young and aged mice

**Fig 4:**
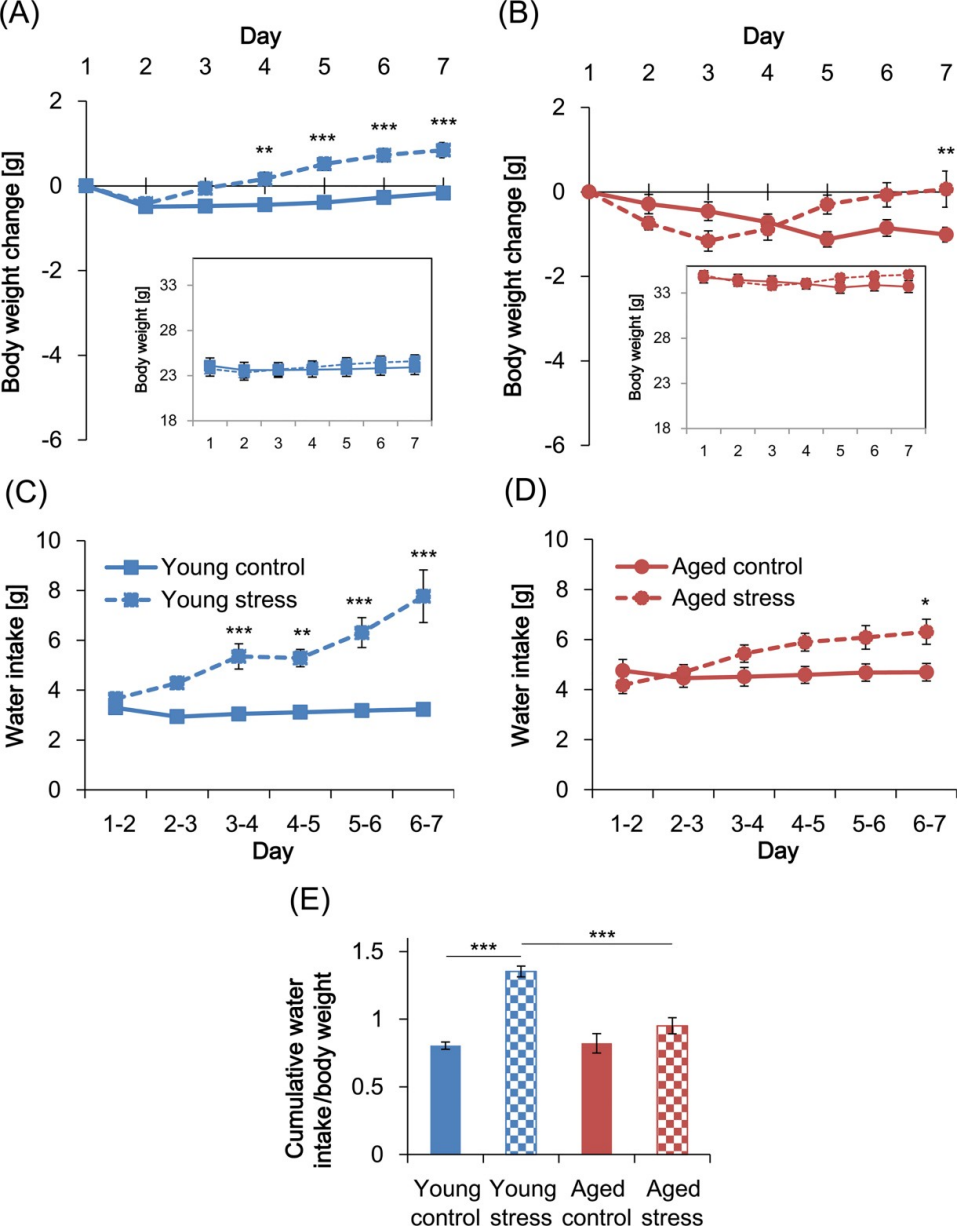
Body weight changes and water intake during the MSDS defeat period. Daily body weight of young (A) and aged (B) mice and daily water intake of young (C) and aged (D) mice data are shown. The inset graphs in (A) and (B) indicate the baseline body weights of the young and aged mice, respectively. The *t* scores for Days 1–7, respectively, were: (A) *t*_294_ = 0.000, 0.4526, 2.603, 3.791, 5.658, 6.224, and 6.309; (B) *t*_280_ = 0.000, 1.408, 2.175, 0.5012, 2.547, 2.415, and 3.33; (C) *t*_252_ = 0.6323, 2.334, 4.007, 3.77, 5.423, and 7.857; (D) *t*_240_ = 1.061, 0.4268, 1.703, 2.404, 2.584, and 2.973. (E) Cumulative water intake per body weight during Days 1–7 is shown. Data are presented as means ± standard error of the mean (SEM) (young control, *n* = 22; young stressed, *n* = 22; aged control, *n* = 20; aged stressed, *n* = 22). *: *p* < 0.05; **: *p* < 0.01; ***: *p* < 0.001 (Bonferroni test). MSDS, mild social defeat stress.

Repeated social defeat stress often causes changes in body weight and liquid intake [14]; however, some studies have shown a reduction in these metabolic parameters in defeated mice while others have shown a gain [7, 14, 15]. Body weight changes were significantly affected by stress in the young stressed mice (2-way repeated measures ANOVA: *F*_1,42_ = 21.59, *p* < 0.001) and time (*F*_6,252_ = 25.04, *p* < 0.001) and there was a significant time × stress interaction (*F*_6,252_ = 14.85, *p* < 0.001) (Fig 4A). Post-hoc comparisons showed that there was a significant increase in body weight in the stressed group compared with the control group after Day 4. The daily water intake by young stressed mice was also significantly affected by stress (*F*_1,42_ = 41.56, *p* < 0.001) and time (*F*_5,210_ = 9.163, *p* < 0.001); also, there was a significant time × stress interaction (*F*_5,210_ = 8.43, *p* < 0.001) with defeat inducing an increase in water intake (Fig 4C).

Neither weight changes nor water intake were significantly affected by stress in the aged mice (2-way repeated measures ANOVA: weight change: *F*_1,40_ = 0.6599, *p* = 0.4214; water intake: *F*_1,40_ = 2.836, *p* = 0.0999) but both were significantly affected by time (weight change: *F*_6,240_ = 5.647, *p* < 0.001; water intake: *F*_5,200_ = 10.25, *p* < 0.001); also, there was a significant time × stress interaction for both parameters (weight change: *F*_6,240_ = 8.792, *p* < 0.001; water intake: *F*_5,200_ = 9.852, *p* < 0.001; Fig 4B–4D). Post-hoc comparisons detected significant differences only on Day 7 in the aged groups (body weight: *t*_280_ = 3.33, *p* < 0.01; water intake: *t*_240_ = 2.973, *p* < 0.05), unlike the young groups in which defeat-induced increases were continuously observed during Days 4–7. To make a direct comparison of water intake between both age groups, intake was normalized to body weight for each mouse and summed over Days 1–7. Two-way ANOVA results indicated significant effects of stress (*F*_1,82_ = 43.8, *p* < 0.001) and age (*F*_1,82_ = 14.15, *p* < 0.001; Fig 4E), with a significant stress × age interaction (*F*_1,82_ = 16.81, *p* < 0.001). Post hoc comparisons revealed that there was a significant defeat-induced increase in water consumption in young mice (*t*_82_ = 7.673, *p* < 0.001), but not in aged (*t*_82_ = 1.76, *p* = 0.1644). There was also a significant difference between the young stressed and the aged stressed mice (*t*_82_ = 5.629, *p* < 0.001). These data suggest that the defeat-induced increase in water intake and body weight during the social defeat period is attenuated by age.

## Discussion

In the present study, we developed a less intensely stressful social defeat paradigm, compared to those used in previous studies, to evaluate how social defeat affects the behavioral phenotypes of young and aged mice. We had 3 major findings: (i) aged mice exhibited social avoidance in a similar way to young mice after social defeat; (ii) unlike young stressed mice, aged stressed mice showed a reduced sucrose preference, suggesting that the reward systems of aged mice may be more vulnerable to stress; and (iii) stress-induced increases in body weight and water intake during the social defeat period were attenuated by age.

During MSDS, the young and aged mice experienced different attack latencies, possibly due to their different body weights (Fig 4A and 4B), since aggressors may be more inclined to initiate attacks more quickly when the intruders are smaller. However, the number and duration of attacks was similar between the 2 age groups, suggesting that the degree of aggression exposure was similar for aged and young mice. Some aged mice died or were euthanized regardless of the exposure to MSDS (Fig 1D), raising a concern that systemic poor health could have affected the behavior of the aged mice. To address this, we included only the mice that survived for 7 days after experiencing MSDS in our analysis. Also, liquid consumption per body weight of each aged group was comparable to that of the young control group both during and after MSDS (Figs 3B and 4E), suggesting that the general health status of the aged individuals analyzed did not decline grossly.

Social avoidance behavior is common in socially defeated mice and is postulated to reflect depression- and/or anxiety-like behaviors [7, 8, 10, 16]. The assessment of avoidance behavior as a barometer of behavioral abnormalities in defeated mice has resulted in the identification of numerous molecules and relevant pathways that underlie susceptibility to stress and the onset of affective disorders [8, 17, 18]. In the present study, both young (8–16-week-old) and aged (24-month-old) C57BL/6J mice exhibited similar social avoidance behaviors after repetitive social defeat (Fig 2B and 2C).

Further research is required to ascertain whether the pathways that underlie avoidance behaviors are similar across age groups. However, it is important to note that social avoidance in this model could be caused by a loss of interest in the social target (i.e., a depression-like state) and/or fear of the aggressor (i.e., an anxiety-like state), so either or both behavioral neural circuits could be invoked. The finding that sucrose preference was reduced only in the aged susceptible mice, as determined by their low SI ratios (Fig 3C), suggests that there may be a difference in the pathophysiological conditions relating to rewards in the different age groups. Therefore, to better characterize the emotional circuits that underlie the avoidance behaviors in each age group, we need to use different social targets, such as amicable male or female mice in place of aggressive mice, since this would allow anhedonia to be differentiated from fear in the stressed mice. It would also be helpful to compare the responses of aged and young mice to various types of drugs, such as antidepressants and anxiolytics.

Although Bonferroni comparisons failed to detect any significant differences between the controls and stressed mice of either age (Fig 2A), MSDS significantly affected the time spent in the IZ and the total distance traveled in the target-absent session, implying that MSDS may strengthen basal anxiety-like responses in stressed mice. This should be assessed in a future study using behavioral tests such as the elevated-plus maze, open-field, and light-dark box tests.

The loss of hedonic behaviors is a core symptom of major depression and is frequently evaluated by the sucrose preference test in validated rodent models [9, 19]. Regulation of the hedonic process in the brain is closely associated with the activity of the nucleus accumbens (NAc), which receives dopaminergic inputs from the ventral tegmental area (VTA). Notably, the spontaneous activation of VTA dopaminergic neurons is reduced by chronic cold stress and the release of dopamine also decreases in the NAc in response to reward stimuli following exposure to chronic mild stress [20, 21]. Moreover, artificial excitation of the VTA by optogenetic stimulation has been shown to reverse the decreased sucrose intake that is observed in mice that have been subjected to chronic mild stress, suggesting that dopaminergic activation in the VTA positively regulates hedonic behavior in rodents [22]. However, a reduction in sucrose preference can also be caused by the hyperactivation of dopaminergic neurons in the VTA after social defeat in a context-specific manner [23]. In particular, phasic activation by optogenetic stimulation of the VTA-NAc pathway induces a reduction in sucrose preference in socially defeated mice, whereas inhibition of the same pathway reverses this effect [23]. These different effects of the VTA on sucrose preference could be explained by the intensity of the stressors, since comparatively mild stressors attenuate VTA activity and more severe stressors increase it [24]. Since the stressors that were used in the present study were mild, we speculate that they were insufficient to activate the VTA and reduce sucrose preference in the young, less vulnerable mice. Also, several lines of evidence have indicated that aging *per se* decreases the contents of dopamine and its receptors at sites that are innervated by dopaminergic neurons, including the NAc, in rats [25–28], and such age-related decreases in dopamine receptors have also been observed in humans, with the neural activity of the NAc during reward anticipation and learning declining with age [29–31]. Therefore, our MSDS paradigm may have induced an anhedonia-like state in aged susceptible mice (Fig 3C), possibly by suppressing the activity of the dopaminergic pathways in the reward system.

Body weight change is a common physiological response to exposure to stressors that is usually expressed as weight loss or a suppression of weight gain during or after the stress exposure. Several studies have suggested that conventional social defeat stress, which is characterized by relatively strong physical attacks, inhibits body weight gain in subordinate mice compared with non-stressed controls [6, 7, 13, 14]. However, in the present study, the stressed young mice consistently exhibited greater weight gain than the control mice after the fourth day of the MSDS period (Fig 4A). Though the difference in weight change in response to stress exposure could be attributed to the experimental conditions, the intensity of the aggression-related stress may also have a significant impact on weight changes in socially defeated mice, with a relatively mild defeat inducing body weight gain [14, 15, 32], as indicated by our results.

Although body weight can be influenced by a variety of metabolic parameters including food intake and calorie consumption in a home cage, defeat-induced weight increase may also be due to a polydipsia-like state in stressed individuals [15], which was also observed in the young and aged subordinates in this study (Fig 4C and 4D). Although the mechanism underlying this polydipsia-like state in socially defeated animals remains unclear, activation of the sympathetic nervous system (SNS) is thought to be associated with an increase in water consumption following repeated stress exposure [33]. The SNS is activated within seconds of being confronted by a threat, including social defeat, to cause a fight-or-flight condition [34–36]. This activation enhances the peripheral production of renin by acting on adrenergic receptors in the kidney, resulting in the upregulation of circulating angiotensin II [36], which is known to elevate the volume of water intake by binding to angiotensin type 1 receptors in the circumventricular organs [37]. Thus, the polydipsia-like state that was observed in stressed young mice may be associated with the activation of sympathetic nerves and subsequent renin–angiotensin activity.

Compared with young mice, the increased drinking behavior of aged mice was attenuated by the exposure to social defeat in the present study (Fig 4B–4D and 4E), implying that they have an altered responsiveness in terms of the abovementioned pathways after social defeat stress. In support of this, aged rodents have been shown to exhibit an attenuated increase in drinking in response to a beta-adrenergic agonist and to angiotensin II compared with younger controls [38, 39]. Furthermore, aged rats exhibited reduced upregulation of plasma renin activity following an air jet stressor compared with younger controls [40]. Together, these findings suggest that the reduced increase in drinking in the aged mice in response to social defeat stress may be associated with an attenuation of the stress-induced activation of the SNS and renin–angiotensin systems. Therefore, considering the roles of these systems in stress-coping and adaptation, the attenuation of increased drinking observed in the aged subordinates may reflect an increased vulnerability to social defeat compared with young mice.

## Conclusions

Aged mice exhibited the same social avoidance behavior as young mice after social defeat. Furthermore, unlike their younger counterparts, aged subordinates clearly exhibited an anhedonia-like phenotype after mild defeat stress, despite both groups experiencing similar numbers of physical attacks. Drinking behaviors also differed between age groups after the defeat exposure. Together, these findings indicate that behavioral phenotypes after social defeat were altered and that stress-related behavioral reactions may have been exacerbated by increased age.
